# “They knew the same struggles”: perceptions of a group coping skills intervention in patients with chronic graft-versus-host disease

**DOI:** 10.1007/s00520-025-09153-x

**Published:** 2025-01-16

**Authors:** Joely A. Centracchio, Daniel G. Yang, Annemarie D. Jagielo, Joseph A. Greer, Areej El-Jawahri, Lara Traeger, Ashley M. Nelson

**Affiliations:** 1 Massachusetts General Hospital, Boston, MA USA; 2 Harvard Medical School, Boston, MA USA; 3 Department of Psychology, University of Miami, 5665 Ponce de Leon Boulevard, Coral Gables, FL 33146 USA

**Keywords:** Qualitative research, Quality of life, Coping skills, Self-management, Graft vs. host disease, Hematopoietic stem cell transplantation

## Abstract

**Purpose:**

Chronic graft-versus-host-disease (cGVHD), an inflammatory condition affecting allogeneic hematopoietic cell transplantation (HCT) survivors, is associated with a range of debilitating physical and psychological sequela. Yet HCT recipients with cGVHD are virtually absent from survivorship intervention research. We conducted a randomized clinical trial to evaluate the feasibility and preliminary efficacy of a multidisciplinary group coping skills intervention (Horizons) tailored to meet these patients’ unique needs. For this follow-up qualitative analysis, we evaluated the perceived impact of the Horizons intervention by the group participants.

**Methods:**

We purposefully selected a subset of Horizons participants (*n* = 19) to complete audio-recorded exit interviews via semi-structured interview guide. We used rapid analysis to characterize participant feedback in three domains: (1) motivations to participate, (2) perceived benefits of participation, and (3) impacts of participation.

**Results:**

Findings highlight participants’ motivations to participate centered on desires to connect with others living with cGVHD and to help future patients. Perceived benefits of participation focused on the following categories: (1) connecting with other survivors, (2) learning about cGVHD, and (3) learning coping strategies to manage specific cGVHD symptoms. Impacts of participation on everyday life variably reflected categories of (1) increased sense of empowerment to contact their care team with questions and concerns, (2) increased support and validation in their struggles with cGVHD, and (3) renewed motivation or progress toward personal and health-specific goals.

**Conclusion:**

Study findings demonstrate participants’ appreciation for a group-based opportunity to connect with others living with cGVHD and strengthen skills for navigating cGVHD challenges. Results support the ongoing need for evidence-based interventions to improve quality of life among HCT survivors.

**Trial registration:**

www.ClinicalTrials.gov, identifier NCT04479995. Date of Registration: July 21, 2020.

**Supplementary Information:**

The online version contains supplementary material available at 10.1007/s00520-025-09153-x.

## Introduction

Graft-versus-host disease (GVHD) is an inflammatory syndrome that attacks organs in the body and is a major cause of morbidity and impaired quality of life (QOL) in survivors of allogeneic hematopoietic cell transplant (HCT) [1–5]. Over half of these survivors develop chronic GVHD (cGVHD), with an onset typically occurring between 3 and 18 months following transplantation [6]. cGVHD can affect any organ, though it predominantly affects the skin, mouth, and eyes [7, 8]. Patients face immense symptom burden, including nausea, diarrhea, and a decline in functional status [9]. The disease follows an unpredictable course, with symptoms categorized as mild, moderate, or severe. Treatment typically involves corticosteroids, which offer limited efficacy and can introduce additional toxicities, further complicating the disease’s uncertain trajectory [3, 10, 11]. Management often requires daily use of systemic and topical anti-inflammatory or other immunosuppressive treatments, but adherence to these treatments is frequently challenging for patients [13].

Psychological distress is common alongside the substantial physical symptoms and functional limitations of this illness, with 50% and 30% of patients with cGVHD endorsing depression and anxiety symptoms, respectively. Such heightened distress further exacerbates QOL impairments [2, 5, 12]. Symptoms, including psychological distress, pain, fatigue, insomnia, and cognitive deficits, are often worse for those living with moderate or severe cGVHD [12]. The numerous daily systemic anti-inflammatory and immunosuppressive medications required to manage cGVHD have mixed efficacy, can lead to complications, and pose substantial adherence challenges [10, 11, 13]. Taken together, these factors contribute to the unpredictable trajectory of this illness and its unrelenting disease course. Self-management, including medication adherence, self-care, and coping, is integral to improving post-HCT quality of life and care. Supportive interventions to assist long-term survivors in coping with the disruptive nature of cGVHD are critically needed.

To address this need, we conducted a single-center pilot randomized clinical trial to evaluate the feasibility and preliminary efficacy of a multidisciplinary group coping skills intervention (Horizons) versus minimally enhanced usual care among 80 adult patients with moderate or severe cGVHD. Participants in the Horizons arm received eight weekly group sessions co-led by a transplant clinician and behavioral health clinician using a videoconferencing platform. The intervention integrated patient education about cGVHD symptoms and treatments with the introduction and practice of skills for coping with everyday cGVHD-related challenges. Results were reported previously and demonstrated the feasibility of study enrollment, session attendance, and study retention, as well as preliminary efficacy for improving quality of life and reducing symptoms of depression and anxiety among participants in the Horizons arm relative to participants in the control arm [14]. The objective of this follow-up study was to identify participants’ perceptions of the Horizons Program impact and value via in-depth exit interviews. We also explored participants’ motivations to participate and recommendations for program improvement. The findings of this work will inform future research to tailor supportive care interventions for post-transplant survivors managing the challenges of cGVHD.

## Methods

We conducted exit interviews with the intervention arm participants in a randomized, nonblinded clinical trial of the Horizons Program (*n* = 39) versus minimally enhanced usual care (*n* = 41) for patients with moderate or severe cGVHD (ClinicalTrials.gov identifier: NCT04479995). The study was conducted at Massachusetts General Hospital Cancer Center (MGH) and approved by the Dana-Farber/Harvard Cancer Center Institutional Review Board.

### Participants

Eligible patients included adults aged ≥ 21 years who underwent allogeneic HCT, were experiencing moderate or severe cGVHD as graded by their transplant oncologist using NIH consensus criteria [15], were currently receiving care at the MGH Blood and Marrow Transplant Clinic, and were able to participate in an English-language intervention. MGH has a survivorship clinic situated within the bone marrow transplant (BMT) service that is comprised of BMT oncologists and advanced practice providers. This specialty clinic focuses on treating post-transplant complications and providing psychosocial support in managing self-care regimens. Patients who met inclusion criteria were eligible regardless of the time elapsed since cGVHD diagnosis. The age criterion was established to focus recruitment among adults who are responsible for their own medical care. We excluded patients with severe comorbid conditions or cognitive impairment that the treating clinician believed would prohibit informed consent or participation.

### Procedures

Study procedures are reported in detail in the primary outcomes paper [10] and are briefly summarized here. Research staff reviewed the transplant clinic outpatient schedule and electronic health records to identify potentially eligible patients and then proceeded to contact the transplant oncologist to confirm moderate or severe cGVHD status and request permission to approach the patients about the study. Study staff approached 119 patients for participation between August 2020 and March 2022, with 73.1% (*n* = 87) providing informed consent, which was obtained verbally over the telephone or in-person during routine clinic visits. Participants completed surveys of primary study endpoints, including quality of life, distress, and coping at baseline and 10 and 18 weeks post-randomization. Upon completion of the baseline assessment, participants were randomly assigned in a 1:1 ratio to receive either the Horizons Program or minimally enhanced usual care. Participants, study staff, and investigators were not blinded to the randomization assignment.

### The Horizons Program intervention

The Horizons Program was based on prior research in HCT recipients [1, 2, 5]; a self-management framework [16]; evidence-based strategies drawn systematically from mindfulness-based (e.g., acceptance), cognitive-behavioral (e.g., behavioral activation), and positive psychology principles (e.g., meaning-making); and feedback from patient/caregiver stakeholder groups [17]. Participants in the Horizons study arm received an intervention workbook and eight weekly 90-min sessions with four to six participants per group, co-led by a transplant clinician (transplant oncologist or nurse practitioner) and behavioral health expert (social worker, psychologist, or psychology fellow) via a secure videoconferencing platform. Facilitators integrated discussion of medical information about cGVHD and survivorship with self-management skills. Time was also allotted for social support and discussion during these sessions. Participants were encouraged to set practice goals between sessions and bring questions about medical history or laboratory tests to their HCT clinicians to reinforce self-management principles and empowerment. Session topics included sharing challenges of life after transplant, managing cGVHD symptoms and quality of life with medications and behavioral strategies, coping with uncertainty, practicing self-care, navigating relationships and intimacy, making meaning from experiences, and setting goals for the maintenance of program skills. Following select sessions with mindfulness-based components, study staff sent electronic files containing audio-recorded mindfulness exercises from the workbook.

### Exit interview

We developed a semi-structured exit interview guide for participants that included questions and probes addressing motivations to participate, program content, perceived benefits, perceptions of the use of videoconferencing for group sessions, and recommendations to improve the program. The interview guide was developed by four authors (L.T. [psychologist, female], A.M.N. [psychologist, female], A.E. [oncologist, female], A.D.J. [research assistant, female]) with experience in qualitative research. The exit interview was added to the protocol mid-way through the study. After the protocol was revised, all Horizons study arm participants were invited to complete the exit interview to ensure the representation of participants who did or did not complete all intervention sessions. We anticipated achieving sufficient redundancy in participant responses after 18–20 interviews [18]. At the 10-week time point, trained study staff members (A.D.J., D.G.Y., E.M.) conducted each exit interview with participants by phone. At the end of each audio-recorded interview, study staff conducted respondent validation by restating a summarized version of the responses and providing an opportunity for clarification to help enhance rigor in subsequent data interpretation.

### Rapid analysis

Our team adapted a rapid analysis approach to characterize participants’ perceived benefits and impacts of the Horizons intervention [19]. This approach involved constructing a template based on the interview questions and making refinements based on a review of the audio recordings. We organized interview questions into three overarching domains and then entered the responses per domain into a matrix. We then synthesized the responses into categories per domain. For instance, there were several questions related to the effects of program participation that were synthesized into the impacts of the participation domain. Key takeaways from questions corresponding to these domains were then entered into a matrix with summaries by person (Respondent X Neutral Domain Header). Two study team members tested the template using the initial interviews conducted to assess the template’s general usability and relevance. Once refined, the staff independently continued summarizing the interview data using the finalized domains and then reviewed together to identify and resolve discrepancies (A.M.N. [psychologist, female], D.G.Y. [research assistant, male], E.M. [research assistant, female]). Within each domain, the study team met to identify categories that captured participants’ comments. Categories were compared with the recordings to ensure they reflected the raw data. The study team extracted illustrative quotes from the audio recordings to highlight specific categories. We utilized non-transcribed audio recordings, which have been found to produce comparable qualitative findings to transcribed audio recordings (e.g., [20, 21]). Furthermore, the analysis of thoughts, opinions, and experiences in a systematic and efficient manner is well-suited for rapid analysis [19]. All data collection was completed prior to data analysis.

## Results

### Participant characteristics

Participant characteristics of the overall Horizons Program sample (*N* = 39) are summarized in Table 1. As previously reported, 32 (82.1%) completed all eight intervention sessions, with only two patients reporting being too ill to participate [14]. Of the 21 participants contacted for the interview, 19 completed the interview from July 2021 to May 2022. The interview duration ranged between 15 and 41 min (*Mdn* = 30). Participant characteristics generally reflected those of the overall parent trial (Table 1). Exit interview participants (age *Mdn* = 64 years, range: 28–79 years; *n* = 13 female; *n* = 15 moderate cGVHD; 19 non-Hispanic White) were a median of 3.5 years (range: 10 months–14 years) post-transplant. Most participants were married (*n* = 14) and had at least 2 years of college education (*n* = 17). Approximately half of the participants were working full- or part-time (*n* = 9), and half were retired or not working due to their illness (*n* = 10).

**Table 1:** Participant demographics

Characteristic	Horizons Program sample (*N* = 39)	Exit interview subsample (*n* = 19)
Age in years (median, range)	64 (28–79)	64 (28–79)
Gender, women	23 (59%)	13 (68%)
cGVHD severity		
Moderate	32 (82%)	15 (79%)
Severe	7 (18%)	4 (21%)
Post-BMT time in years	3.09 (10 months–14 years)	3.54 (10 months–14 years)

*cGVHD*, chronic graft-versus-host-disease; *BMT*, bone marrow transplant

### Summary of domains and categories

We identified results in three major domains capturing participants’ experiences of the Horizons Program: (1) motivations to participate, (2) perceived program benefits, and (3) perceived impacts of participation. The domains and their respective categories are described below (see also Tables 2, 3, and 4 with representative quotes).

**Table 2:** Categories in *motivations to participate* domain (*n* = 19)

Categories	Example quotes
Desire to connect with others	*[My primary reason for joining] was to get information on what other people are dealing with, how they’re dealing with it. Especially graft-versus-host stuff. And to meet some other folks who are going through the same stuff.* (ID 73, Male, Age 64, Moderate GVHD)
	*I had been dying to actually be in one of these groups. I think all of us in this particular group wanted to talk to people who had similar BMT issues to what we had.* (ID 83, Male, Age 69, Moderate GVHD)
Desire to help future others	*Just to help out. I like to be able to give back to people like [research staff] and my caregivers. So when [research staff] approached me about needing people for a study, it was kind of an immediate no-brainer. It’s one of the things that helps me cope, I think.* (ID 67, Female, Age 36, Moderate GVHD)

**Table 3:** Categories in *perceived program benefits* domain (*n* = 19)

Categories	Example quotes
Connecting with other survivors during sessions	*Meeting new people with GVHD because I didn’t know anybody who had it…that was the most important thing. There were five of us and I think all of us [in the group] kind of kept reaffirming that we have figured out how to move on with life and it’s doable. It can be done. Don’t give up.* (ID 53, Female, Age 71, Moderate GVHD)
Learning about cGVHD	*I did learn about a few different things that I hadn’t known before. One of the [participants] had that cauterizing done in her eyes, and I was really scared to do it. She gave me a lot of information which was really helpful.* (ID 46, Female, Age 62, Moderate GVHD)
Learning about coping strategies	*I learned some things about myself. If you asked me if I was gonna enjoy that, I would have said absolutely not. But it was great…. I try to do the whole mindfulness things when I’m in bed. So yeah, for sure it’s helped me.* (ID 64, Male, Age 69, Moderate GVHD)

**Table 4:** Categories in the *impacts of participation* domain (*n* = 19)

Categories	Example quotes
Increased sense of empowerment to contact care team with questions and concerns	*It was enlightening for me because a lot of the things that I don’t think of as GVHD, you know, could be. It was nice to know that it might not just be getting old. One of the best parts of the program was that you encourage people, even if it’s something minor, to reach out to your doctor and to say, ‘is this possibly GVHD?’* (ID 72, Female, Age 77, Moderate GVHD)
Increased support and validation	*I’m very happy that I took part in this. I’m extremely happy that I got to know other people. I’m extremely grateful that I got to share things with people that really understand. Absolutely invaluable. That’s the best part.* (ID 64, Male, Age 59, Moderate GVHD)
	*Even if they [other participants] didn’t have the same symptoms, they knew the same struggles … Everyone thinks ‘oh, you’re in remission.’ You know, ‘your cancer’s gone.’ But they don’t understand the things you carry with you like how tired you can get, or your eyes bothering you, or just the other things that go with it.* (ID 86, Female, Age 56, Moderate GVHD)
Renewed motivation toward personal and health-specific goals	*I get myself in trouble with not taking my pills on time. And I’ve been trying really hard to not let that happen to me. Knowing what time the meeting started was good for me because when the meeting was over, it was time for me to take my pills. So, I hadn’t been missing any of it, you know, which was really really good. So that kind of helped me like that. Now I’m back on track again.* (ID 46, Female, Age 62, Moderate GVHD)
	*After I saw how active everybody was […] I was kind of pushing myself to do more. My friend just said to me ‘where’s all this energy coming from?’ and I go ‘I don’t know’ [laughs]. She goes, ‘I think it’s because you’ve been doing stuff.’ … They [the group participants] were great.* (ID 50, Female, Age 59, Severe GVHD)

#### Domain 1: Motivations to participate

Participants reflected that at the time of study enrollment, their motivations to participate centered on desires to (1) connect with others living with cGVHD who may be facing similar challenges, (2) “give back” to their transplant center by participating in the study, and/or (3) help future patients by sharing their own experiences. Participants commonly viewed the Horizons Program as a novel and desired setting to engage with fellow survivors and, through their participation in this research, an opportunity to inform and improve supportive care interventions for this population.

#### Domain 2: Perceived program benefits

Perceived program benefits focused on (1) connecting with other survivors and (2) learning about cGVHD and coping strategies to manage specific symptoms. This domain highlights components of the program that were enjoyable and useful to participants. Participants commonly felt that Horizons was a valuable and comforting opportunity to build community with their peers in the context of a socially isolating medical condition. Many respondents appreciated the opportunity to meet and connect with other cGVHD survivors with shared experiences. Though some participants faced initial trepidation about establishing rapport with the group, they noted that compassion from group facilitators and other group members helped them to grow more comfortable in subsequent sessions. Reflective of some of the social bonds created in this program, several participants voiced a desire to check-in with fellow group members following the completion of the Horizons eight group sessions.

When asked specifically about their experiences learning more about cGVHD, participants commonly highlighted the importance of being informed of the diverse range of cGVHD symptomatology. Given participants’ perceptions of GVHD as “a field of unknowns,” many participants found the education to be useful, with descriptions such as “enlightening,” “inclusive,” and “invaluable.” Yet, some participants would have preferred a greater emphasis on information specific to cGVHD symptom management whereas others felt they were already familiar with the clinical content. Coverage of sexual health and intimacy was variably perceived as a valuable and challenging, sometimes uncomfortable, discussion topic in the group setting.

Many participants also reflected on the utility of their newly acquired coping strategies. Some individuals were initially wary of techniques like mindfulness and meditation; for these participants, practicing these techniques was surprisingly helpful, enjoyable, or effective despite these hesitations. Furthermore, there was a newfound sense of awareness of relevant strategies, support networks, and resources shared by peers and co-facilitators alike. The program’s discussions on navigating help-seeking and maintaining self-care activities made such tasks feel more feasible amidst current demands.

#### Domain 3: Impacts of participation

Impacts of participation on everyday life variably reflected (1) an increased sense of empowerment for participants to contact their care team with questions and concerns, (2) increased support and validation in their struggles with cGVHD, and (3) renewed motivation toward personal and health-specific goals. This domain captures takeaways and reflections on the overall influence of the program on participants’ lives.

In learning about cGVHD symptoms, participants felt more empowered to contact their care team with their questions or concerns “even if it was something minor” and ask, “Is this possibly GVHD?.” Several participants planned to prioritize ongoing communication with their medical providers. The perspectives shared in the group normalized many of their experiences, which bolstered their confidence to proactively address their needs at upcoming appointments. Participants also commonly found the emotional support during group sessions to be invaluable, particularly in improving their overall mood. They noted that their peers’ stories of transplant journeys and challenges resonated deeply. One participant noted that during group sessions, they shared things for the first time “that [they] just could never share with anybody before” as they finally felt comfortable and safe to do so in this forum. Participants further reflected that program sessions helped them get back on track with specific goals, such as reestablishing or making progress in cGVHD self-care and medication adherence, physical exercise, and pleasurable activities.

### Challenges and recommendations

Participants described notable challenges or recommendations for improvements across the Horizons Program timing, structure, content, materials, and delivery.

- **Timing: **Some participants noted that they already had some years of experience managing cGVHD and that program benefits may be increased if the Horizons Program were offered sooner after their cGVHD diagnosis. Similarly, a few participants proposed dividing the groups into individuals who are more newly diagnosed and those who are further out from diagnosis.
- **Structure: **While several participants valued how the program structure helped the group maintain focus during the sessions, a few participants felt that the program should have more time for group members to interact with each other or more flexibility to pivot to spontaneous topics that participants raise during sessions. Overall, participants commonly felt that the program was brief in length and that one to two additional sessions would be desirable.
- **Content: **Individual participants suggested other tailored topics for program content, including clinical trial navigation and literacy, relationships with caregivers and transplant donors, referrals to specialists and other resources, fertility, pain management, and future planning or prognosis. One participant further suggested having a knowledgeable HCT survivor serve as an additional group facilitator. When probed more specifically about whether they would be interested in including their caregiver in the program, responses were varied (*n* = 6 participants were interested, *n* = 8 not interested, *n* = 2 mixed, *n* = 3 no response). Some participants appreciated the focus of a patient-oriented group that allowed them a unique supportive space to discuss patient-specific perspectives independent from their caregivers. Those interested in incorporating caregivers suggested that the program may be valuable in facilitating caregiver knowledge and understanding. Two participants recommended a separate, caregiver-specific coping group to allow for adequate time devoted to their concerns.
- **Materials: **Program materials were variably useful between sessions. Participants commonly found that the program workbook helped them prepare and set expectations for each session. In contrast, participants generally did not use the audio files of meditation exercises between sessions.
- **Delivery:** The telehealth modality was a surprisingly positive experience for many participants, offering the ability to join the sessions from a comfortable setting without requiring travel. However, several participants felt that an in-person format would have made the experience more personal. Upon probing, only one participant would have been interested in attending this program via telephone.

## Discussion

This qualitative analysis synthesizes in-depth exit interview feedback from the first study to empirically test a multidisciplinary supportive care intervention for patients living with cGVHD. The aim of this analysis was to identify themes about the Horizons Program impact and value as perceived by Horizons participants. The present findings highlight the perceived importance of a group-based opportunity to connect with others living with cGVHD and strengthen skills for navigating cGVHD-related challenges.

Among participants, connecting with other people living with cGVHD was both an initial motivation to participate in the Horizons trial and a subsequent perceived benefit of program participation. Qualitative work has elucidated that disease-related social isolation is a critical concern in this population, given the protracted HCT hospitalization period and subsequent immune vulnerability that limits social engagement in public settings for many survivors [17, 22–24]. Group support was perceived as a fundamental program component facilitating meaningful connections and a sense of community among group members. Initial discomfort with the group setting among some participants highlights the potential value of the Horizons Program co-leadership model wherein the transplant and behavioral health clinicians can work in tandem to normalize survivorship experiences as well as foster group cohesion among participants with diverse cGVHD experiences, personal backgrounds, interpersonal styles, and goals for participation. Research by Siwik et al. [25] underscores the strengths, acceptability, and feasibility of a multidisciplinary cancer survivorship group-based intervention framework, including the streamlining of survivorship care across a breadth of topics.

While participants were not primarily motivated to participate in Horizons to learn new skills, results indicated that skill acquisition was subsequently perceived as a key program benefit. This finding emphasizes the value of addressing coping skills and cGVHD self-care, even among participants who felt they had sufficient knowledge about managing cGVHD-related challenges prior to the Horizons Program. Moreover, the program allowed group members to share successful self-care and symptom-management strategies with peers who had experienced similar stressors or symptoms. As such, while some participants felt that the program might be most useful soon after cGVHD diagnosis, the benefits of participants both providing and receiving information suggest an advantage of not restricting enrollment based on time since diagnosis. Because many patients often feel isolated from their providers in the survivorship period, enrollment to the Horizons Program across the survivorship period should be flexible and suited to patients’ needs and circumstances [26, 27].

In prior qualitative studies [17, 22], patients shared that they gained control over cGVHD via information seeking and developing a greater understanding of their disease. Our findings also suggest that psychoeducation about the range of potential cGVHD symptoms and skills for coping with cGVHD challenges was useful. Moreover, it was acceptable and tolerable for individual participants to learn about cGVHD symptoms that they may not yet have personally experienced and symptoms such as sexual health problems that they otherwise might be uncomfortable discussing. While sexual health may be a difficult conversation for patients and providers to navigate, participants noted that they were willing to learn about and discuss sexual health concerns.

The Horizons Program had variable impacts on participants, including an increased sense of empowerment to ask questions and address concerns with their care team, increased support and validation related to their cGVHD-related struggles, and/or a renewal of their motivation or progress toward individual goals. Participants’ shared experiences, insights, and recommendations helped to facilitate meaningful discussions about predictable and unpredictable duration, course, and nature of cGVHD symptoms. Informed discussions about cGVHD symptoms are critical to helping patients identify controllable versus uncontrollable aspects of cGVHD and to guide effective self-care strategies (e.g., taking steps to improve controllable symptoms). Among chronic illnesses, a sense of control regarding disease management is a vital factor in successful adaptation [28]. The sense of optimism, hope, purpose, and altruism experienced in the group setting reinforces the power of participants’ sharing of stories and information [29].

Horizons groups inevitably consist of members with limited cGVHD experience as well as those who have endured cGVHD for several years. Future work should investigate whether introducing this program at the onset of the disease trajectory may improve patient-reported outcomes (e.g., quality of life, mood, self-efficacy) longitudinally. Moreover, the Horizons Program content may be well-suited for all survivors of allogeneic HCT during the initial months post-transplant. In fact, many patients report feeling unprepared for the extent to which GVHD impacts functioning and daily living [23]. Grouping participants by length of cGVHD diagnosis is desirable for some, yet it may hinder the sharing of helpful coping strategies and resources that more experienced participants can offer to the group. It may also be worthwhile to allow for more flexibility in the structure and content of the program and incorporate additional opportunities for discussion to enhance group member cohesion and satisfaction. Delivery of a group-based coping intervention is challenging as co-facilitators strive to strike a balance between intervention fidelity and tailoring conversations toward the unique interests and needs of the group. Nonetheless, group interventions are an important forum for fostering support and interaction among individuals living with cGVHD [17, 23, 24].

Program workbooks should be adopted in future iterations of interventions as they aid in preparing for each session’s material and expectations of participants. It is unclear if audio files of exercises reviewed during sessions are beneficial. Consistent with the findings of other telehealth group interventions, conducting Horizons sessions via Zoom is perceived as comfortable and convenient by the majority of participants (e.g., [30]). Some participants still desire an “in-person” approach as they feel they are more conducive to formulating personal connections. Adaptations of this intervention could incorporate an optional in-person session to accommodate such preferences.

Results should be interpreted with attention to study limitations. First, this study was conducted at a single cancer center with limited sociodemographic diversity and all exit interview participants were non-Hispanic White. Findings therefore may not relate to individuals from diverse backgrounds. Future work should expand to transplant centers serving patients with diverse racial and ethnic backgrounds to elevate underrepresented perspectives in the GVHD community. Secondly, this sample was recruited from an academic medical center with a transplant survivorship clinic. Therefore, results may not reflect the preferences of HCT survivors without access to this type of survivorship care. Since exit interviews were added to the study midway during the study, perceptions of the intervention do not reflect the views of all groups conducted. The data were also elicited from participants in a randomized controlled trial and may not consider the perspectives of patients who would have declined trial participation. Additionally, age-related experiences of the program were not specifically probed. Finally, because the analysis used non-transcribed audio files, our ability to cross-check findings may have been hindered by the reliance on such.

Participation in a multidisciplinary group intervention study was perceived as a valuable opportunity for patients with cGVHD to enhance social support, self-efficacy for navigating cGVHD management, and motivation for self-care goals. Taken together, findings support the need for increasing the evidence base for supportive care interventions to improve QOL among patients living with cGVHD.

## Supplementary Information

Below is the link to the electronic supplementary material.

Supplementary file1 (DOCX 16 KB)

## Data Availability

The dataset generated during and/or analyzed during the current study are available from the corresponding author on reasonable request.
