# Supplementary material for: “They knew the same struggles”: perceptions of a group coping skills intervention in patients with chronic graft-versus-host disease
Source: Support Care Cancer. 2025 Jan 16;33(2):102. doi: 10.1007/s00520-025-09153-x (PMC11735584; doi:10.1007/s00520-025-09153-x)
Supplement: Supplementary file 1 — Supplementary file1 (DOCX 16 KB) [file 520_2025_9153_MOESM1_ESM.docx]

**Supplemental Material**

**Table 1.** Example of Rapid Analysis Synthesis from the *Impacts of Participation* Domain.

| **Questions from Exit Interview** | **Rapid Analysis Matrix Heading** | **Domain** | **Categories** |
| --- | --- | --- | --- |
| Do you think being in the study contributed to any changes in your confidence in your ability to manage your graft-versus-host disease symptoms?  Do you think being in the study contributed to any changes in taking your medications as prescribed? Why? *[can provide example, e.g. using any mouthwash/eyedrops etc. as prescribed…]*  Do you think being in the study contributed to any changes in your physical symptoms (e.g., fatigue or sleep) or GVHD symptoms? Why?  Do you think being in the study contributed to any changes in your mood? Why?  Do you think being in the study contributed to any changes in your feeling supported by others? Why?  Do you think being in the study contributed to any changes in your confidence in your ability to manage the demands of living with graft-versus-host disease?  What felt most important to you about your participation in the Horizons program? | Effect on Patients’ Lives | Impacts of Participation | Increased Sense of Empowerment to Contact Care Team with Questions and Concerns  Increased Support and Validation  Renewed Motivation Toward Personal and Health-Specific Goals |
